# Regional health-care inequity in children’s survival in Zhejiang Province, China

**DOI:** 10.1186/s12939-016-0470-1

**Published:** 2016-11-17

**Authors:** Weifang Zhang, Dingwan Chen, Huan Zhou, Yanhua Xu, Zhuopu Xu, Ying Ying, Zhengyan Zhao

**Affiliations:** 1 Department of Administration, Children’s Hospital, Zhejiang University School of Medicine, Hangzhou, 310003 China; 2 Department of Public Health, Zhejiang Medical College, Hangzhou, China; 3 Vaderbilt University Law School, Nashville, USA; 4 Department of Child Healthcare, Yongkang Women and Children’s Hospital, Yongkang, China; 5 Department of Child Healthcare, Children’s Hospital, Zhejiang University School of Medicine, Hangzhou, China

**Keywords:** Health-care inequality, Survival, Children, Mortality rate, Per capita GDP

## Abstract

**Background:**

China is now under a period of social transition, and inequity is evident in the field of health care. We aimed to investigate regional health-care inequalities in children’s survival in Zhejiang Province, China.

**Methods:**

In our study, monitoring data of Zhejiang Province from 2005 to 2014 was collected. The flow of data collection of community-district-city for urban areas or village-township-county rural areas was followed. The factors affecting equity was analyzed including regional economical level and household registry. We adopted standard measures of concentration curve and concentration index to evaluate degree of income-related inequity and the trend of mortality changes.

**Results:**

From 2005 to 2014, overall mortality rate in children under five decreased, and regional disparity reduced markedly, and with a reduced disparity of mortality rate among children from urban and rural areas. In 2014, the mortality rate in children from urban and rural areas was similar. In contrast, the mortality rate in the children from migrant population was more than two folds of that in the children from native residency (7.82 ‰ vs. 3.89 ‰). The mortality rates of newborns (rs = −0.396, *P* < 0.001), infants (rs = −0.553, *P* < 0.001) and children under five (rs = −0.568, *P* < 0.001) were all negatively correlated with per capita GDP in different regions. CI in the newborns, infants and children under 5 years was −0.105, −0.107 and −0.118, respectively. The concentration curve was near to equity curve. The concentration curve was near to equity curve. The mortality rate of children was negatively related with economical level in this study.

**Conclusions:**

The survival status was near to equity. Regional economical development can improve children’s survival but it was not the only social determinant. Migrant population will be the future monitor focus for reducing disparity on healthcare and increase equity in children’s survival.

## Background

Children’s health status is an important index revealing the health conditions of countries and areas. Children’s survival, protection and development are the prerequisite concerns globally. The mortality rates of infants and of children under the age of five are the key indexes to reveal the health status and social development of a country. Macinko et al. [1] revealed that the burden of poor health occurs differently in populations under different social-economical levels and that people of a lower socio-economic status may have worse health outcomes.

The global burden of childhood mortality, morbidity and under nutrition is increasingly focused in the most deprived and underserved populations within countries, partly as a result of inequitable coverage of key maternal and child health and nutrition interventions [2–6]. Duke et al. found under 5 mortality rates in the developed countries of the pacific (Australia and New Zealand) are far lower than the other pacific island developing nations [7]; they found there is a need to know the specific issues to close the gaps in children health. Carrera et al. [8] indicated that a timely and systematic assessment of prevailing health inequality should facilitate equity-enhancing policy and governance, therefore, should receive serious attention in developing countries.

In the past decades, China has carried out many progresses in improving maternal and Children’s healthcare, reducing childhood mortality and increasing children’s survival and nutrition. However, China is now under a period of social transition, with increasingly prominent social problems such as disparity between the rich and the poor, inequity of allocation and opportunities, and inequity is evident in the field of health care. Studies on some developing countries such as Brazil, Chile, Mozambique, India, Indonesia and Nigeland [9–15] have shown that a reduction of both overall child mortality and inequities is possible; reducing inequities will be benefiting for reducing children’s mortality.

The impact of social-economical level on children’s health equity has attracted more and more attention. In this study, we studied surveyed samples from Zhejiang Province, China. Zhejiang Province, one of the most commercial and richest provinces in China, includes 90 counties in 11 regions [16]. We aimed to study the regional health-care inequalities in children’s survival in Zhejiang Province and the impact of social-economical level on children’s health conditions; therefore, policies to improve children’s survival status can be proposed based on the results of this study.

## Methods

This epidemiologic study was approved by Ethical Committee, Children’s Hospital, Zhejiang University School of Medicine (IRB no. 2013118.). The surveillance program in children’s mortality rates was implemented in Zhejiang Province since the 1990s. During the past two decades, a three-level maternal and child health care network (province level-city/district level-county level) has been developed and an integrated child mortality surveillance system has been established with a coverage of 30 sites out of 90 counties in 11 cities or regions (Hangzhou, Ningbo, Wenzhou, Jiaxing, Huzhou, Shaoxing, Jinhua, Quzhou, Zhoushan, Taizhou and Lishui) of Zhejiang Province [16].

In our study, monitoring data of Zhejiang Province from 2005 to 2014 were collected. Data collection was done by well-trained staffs of local maternal and child health care facilities. These data were then reported to higher-level surveillance specialists. The flow of data collection of community-district-city for urban areas or village-township-county rural areas was followed. All information was reported and gathered to the Province Surveillance Office, Children’s Hospital, Zhejiang University School of Medicine [16]. The factors affecting equity was analyzed including regional economical level and household registry. All the regions were stratified into three tiers based on regional per capita GDP. We adopted standard measures of concentration curve (CC) and concentration index (CI) to evaluate degree of income-related inequity and the trend of mortality changes. CI was built based on the social economical level [17]. The CI is defined as twice the area between the CC and the line of equality. Thus, when CC coincides with the line of equality, the CI equals 0. CI is between +1 and −1, with a positive (negative) value suggesting pro-rich (poor) distribution [18, 19]. CI was calculated as $$ \begin{array}{c}\hfill \mathrm{S}=\frac{1}{2}{\displaystyle \sum_{\mathrm{i}=0}^{\mathrm{n}\hbox{-} 1}\left({\mathrm{Y}}_{\mathrm{i}}+{\mathrm{Y}}_{\mathrm{i}+1}\right)}\left({\mathrm{X}}_{\mathrm{i}+1}\hbox{-} {\mathrm{X}}_{\mathrm{i}}\right)\hfill \\ {}\hfill \kern1.5em \mathrm{C}\mathrm{I}=2\times \kern1em \left(0.5\hbox{-} \mathrm{S}\right)\hfill \end{array} $$.

All statistical analysis was performed using SPSS 16.0, taking the sample design into account.

## Results

### The trend of mortality rate reduction in children under five

**Fig. 1:**
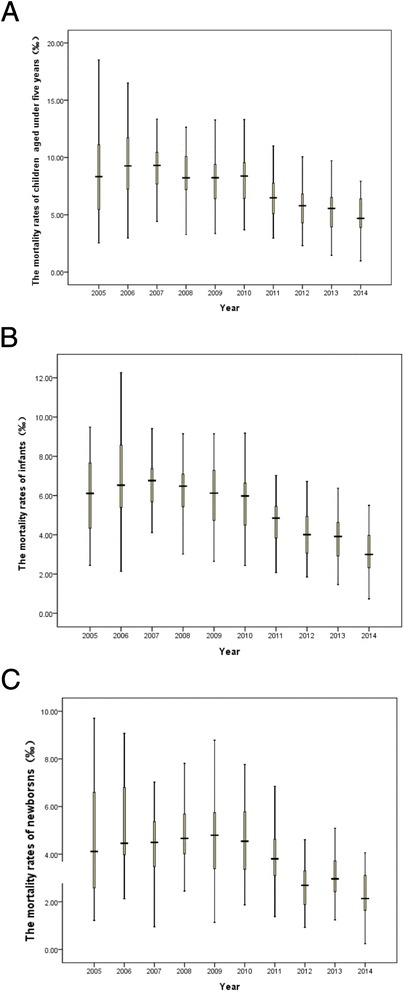
The trend of mortality rate reduction in children under five (**a**) and infants (**b**) and newborns (**c**)

**Fig. 2:**
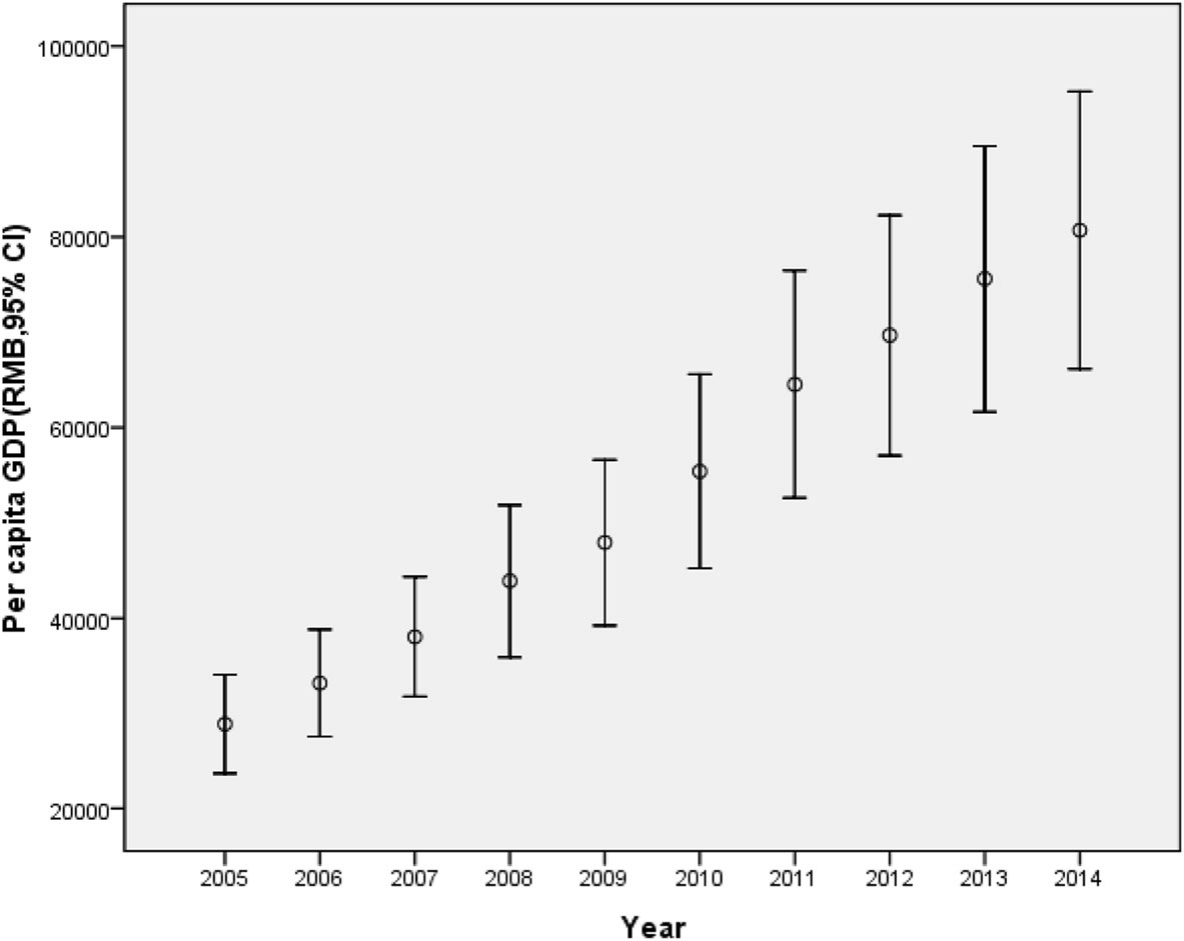
Per capita GDP in the studied regions increased by years, and with increased regional disparity

From 2005 to 2014, overall mortality rate in children under five decreased, and regional disparity reduced remarkably. The trend of reduction was significant in infants and children under five (Fig. 1). Mortality rate in the newborns was floating, but the overall decreasing trend can be observed obviously (Fig. 1c). Per capita GDP in the studied regions increased by years, and with increased regional disparity (Fig. 2).

### The trend of mortality rate reduction in children with different household registry

**Fig. 3:**
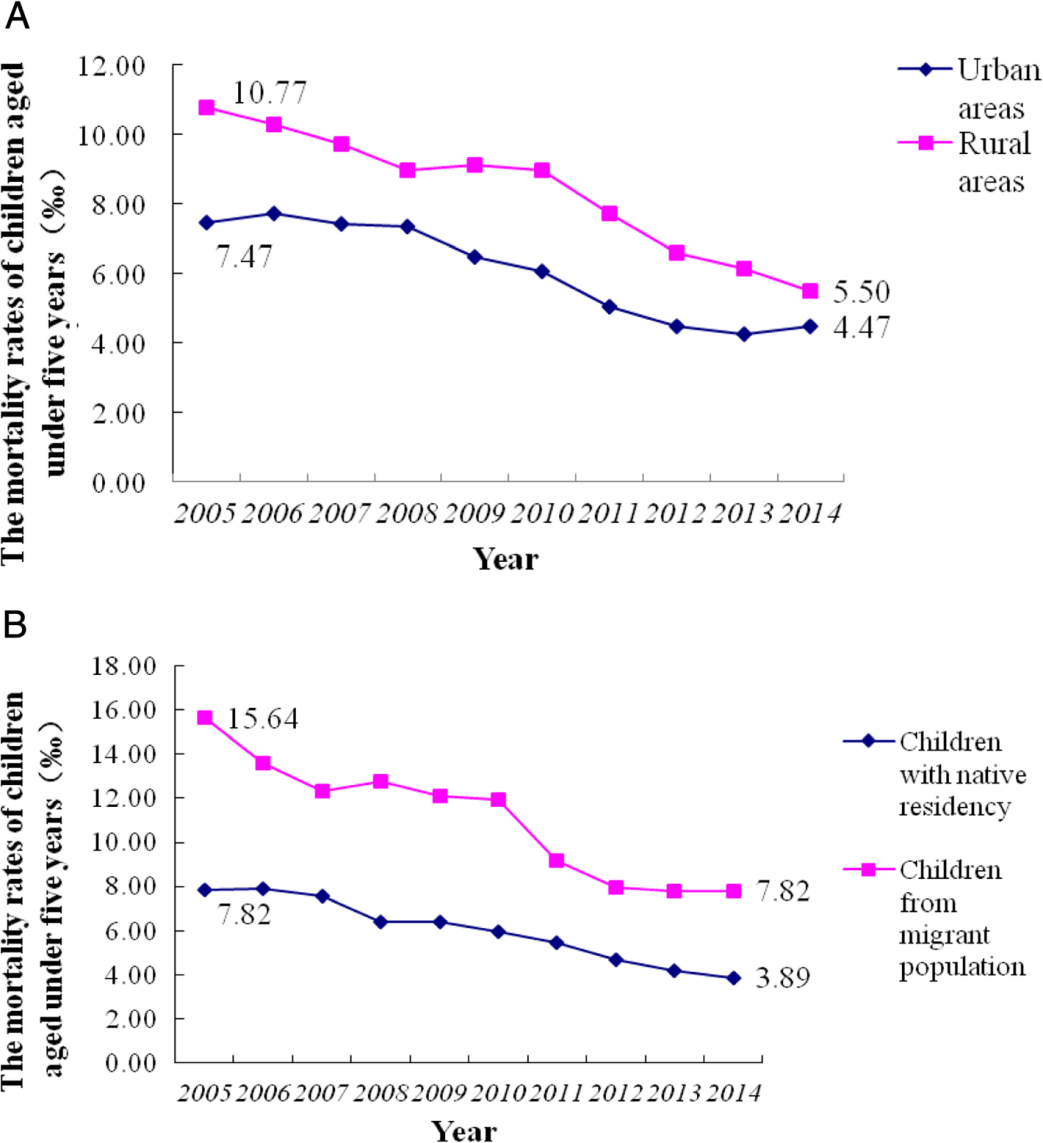
The disparity of mortality rate between urban and rural areas (**a**) and between children with native residency and from migrant population (**b**)

The children were separated into different groups based on different household registry: urban area and rural area; native residency and migrant population. From 2005 to 2014, children’s overall mortality decreased, with a reduced disparity of mortality rate among all children (urban area and rural area; native residency and migrant population). The disparity of mortality rate between urban and rural areas decreased most remarkably (Fig. 3a). In 2014, the mortality rate between in children from urban and rural areas was similar. In contrast, a significant difference was found between the children with native residency and migrant population (Fig. 3b); the mortality rate in the children from migrant population was more than two folds of that in the children from native residency (7.82 ‰ vs. 3.89 ‰).

### The relation of children’s mortality rate and regional economical level

**Fig. 4:**
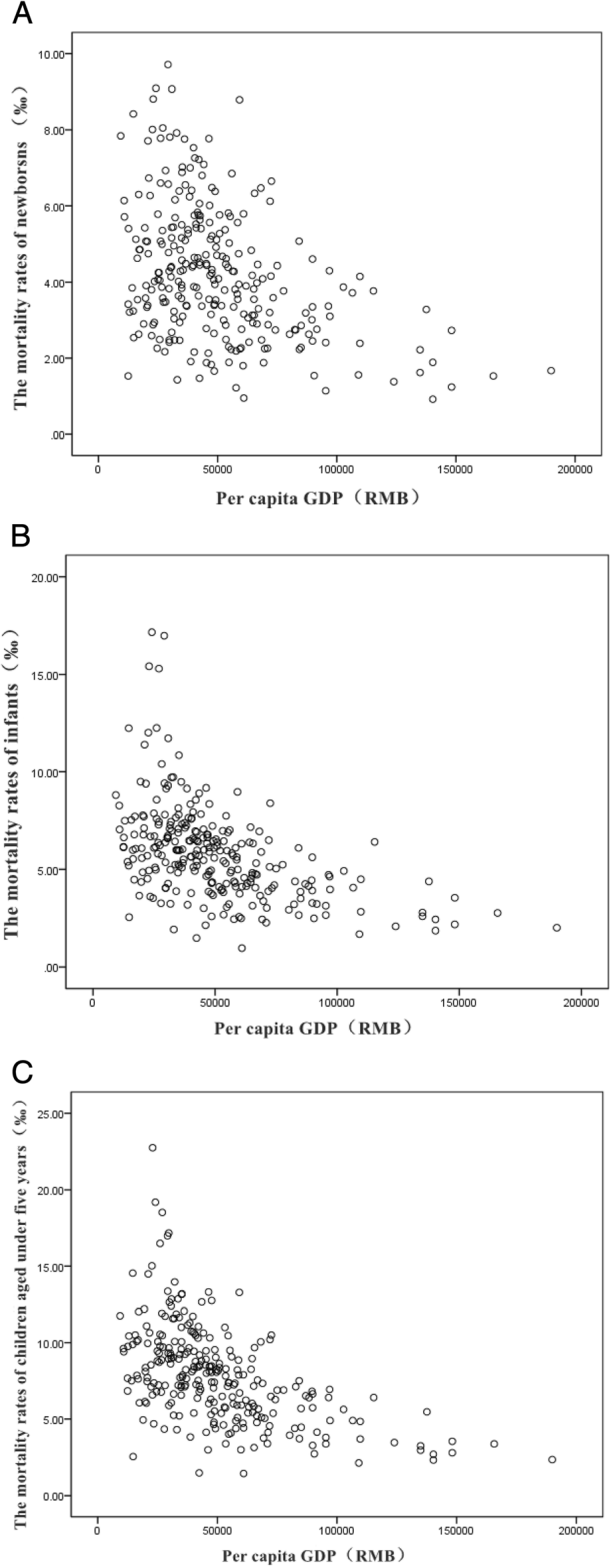
The mortality rates of newborns (**a**), infants (**b**) and children (**c**) aged under 5 years were negatively correlated with per capita GDP in different regions during 2005 and 2014

For exploring the relation of children’s mortality and economical level, we ploted scatter diagrams based on the mortality rate in children under five and mean regional per capita GDP during the 10 years from 2005 to 2014. The mortality rates of newborns (rs = −0.396, *P* < 0.001), infants (rs = −0.553, *P* < 0.001) and children under five (rs = −0.568, *P* < 0.001) were all negatively correlated with per capita GDP in different regions (Fig. 4a–c).

**Fig. 5:**
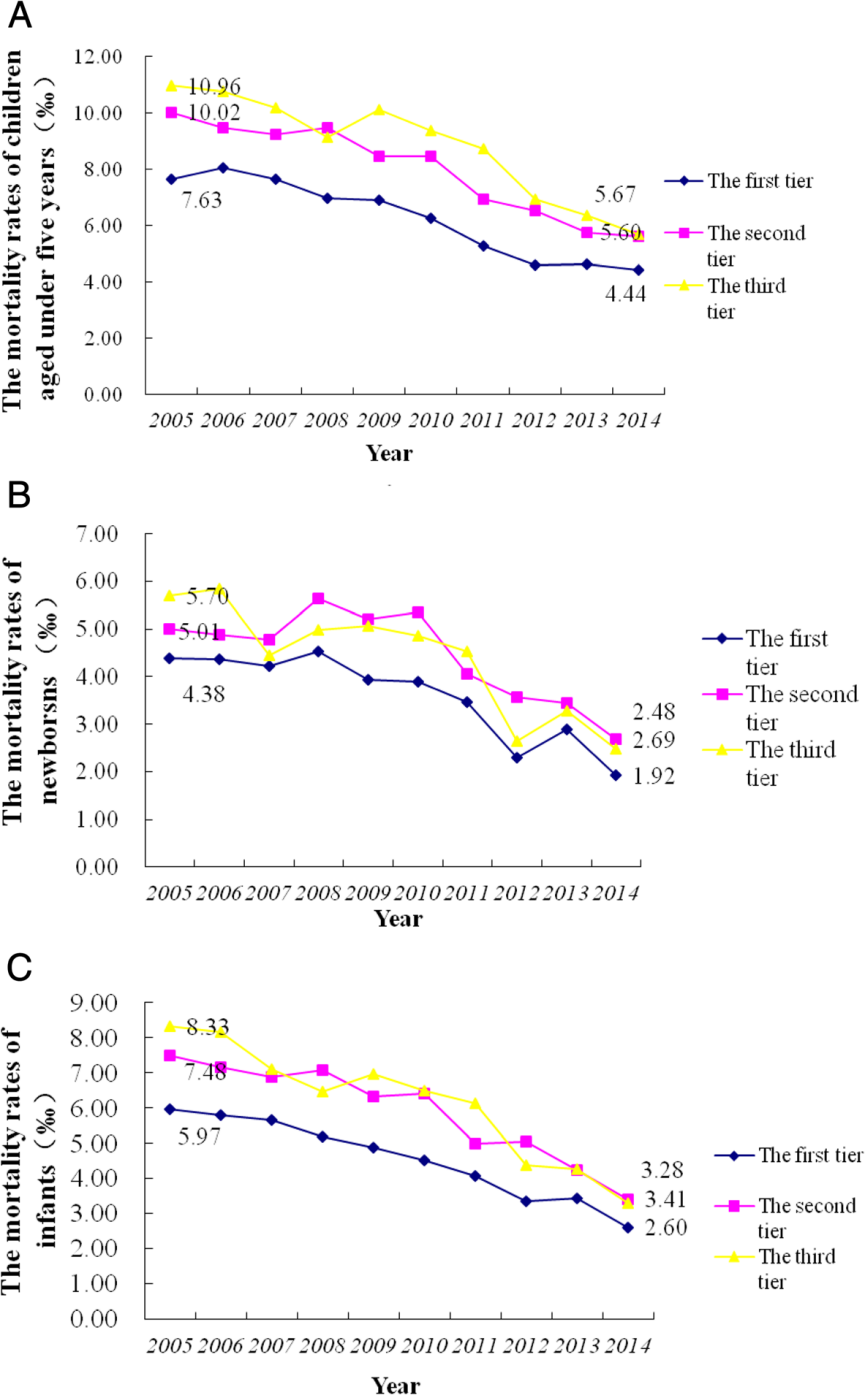
The trend of mortality rate in the three tiers for children under five (**a**), newborns (**b**) and infants (**c**)

For surveying children’s survival equity, we also stratified the 30 sampled regions into three tiers based on mean per capita GDP from 2005 to 2014. Mortality rates of newborns, infants and children under five reduced in all the 30 regions. The regions in the third tier (with low economical level) reduced most remarkably, and the regional difference of children’s mortality rate reduced gradually. The difference of mortality rate among the three tiers was still very significant; overall mortality rate of children under five in the first tier was significantly lower than those in the other two tiers (Fig. 5). Mortality rate of all children under five including neonates and infants in the second and third tier was overlapping which indicating other factors may affect the mortality rate of children in these two tiers.

### Equity analysis of children’s survival level

**Fig. 6:**
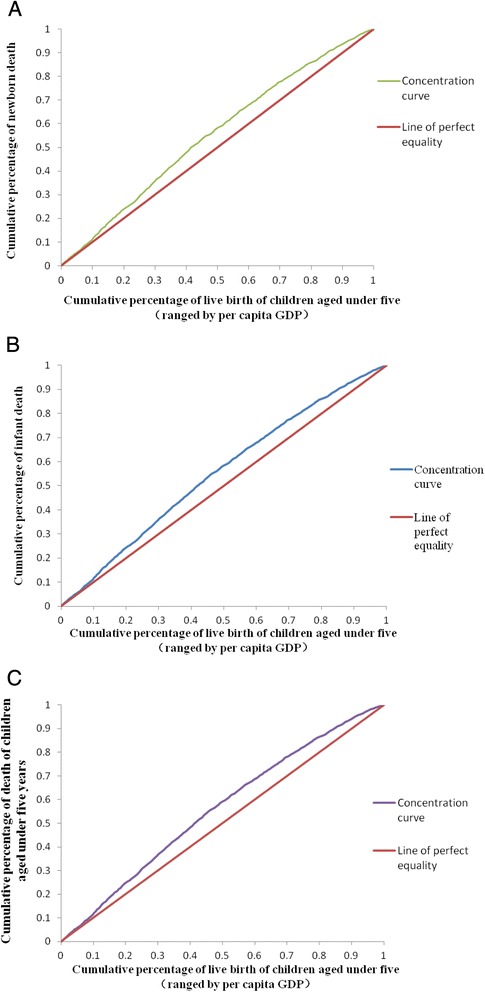
Concentration curve in the newborns (**a**), infants (**b**) and children under five (**c**)

The concentration curve and concentration index (CI) were two commonly used indicators for indicating health equity, which can reveal the relation of regional economical level and health indexes accurately. CI was more sensitive to indicate the social-economical effect and most used in health economics. If CI is negative, which means that children’s deaths concentrate in the regions with low per capita GDP; Children’s mortality rate is much higher in the regions with much lower GDP; high CI value means severe inequity in the survival level. CI in the newborns, infants and children under 5 years was −0.105, −0.107 and −0.118, respectively. The concentration curve was near to equity curve. Our results revealed that the mortality rate of children was negatively related with economical level (Fig. 6).

## Discussion

Under-five mortality is related to many factors. This study analyzed three factors including per capita GDP, residence (urban or rural area) and native residency or migrant population; we aimed to investigate effects of social-economical level on children’s mortality.

### Improved equity of children’s survival in urban and rural areas

Studies on difference of children’s mortality have been carried out for many decades [16, 20, 21]. Boutayeb A reported that mortality under five in Morocoo urban and rural regions reduced dramatically from 38 and 69 ‰, respectively in 2004 to 25.4 and 35.1 ‰, respectively in 2011; the difference reduction in rural regions was larger than that in urban regions [22]. Our surveillance data in Zhejiang Province revealed that in urban regions mortality of children under five reduced from 7.47 ‰ in 2005 to 4.47 ‰ in 2014 (unpublished data); in rural regions, mortality of children under five reduced from 10.77 ‰ in 2005 to 5.50 ‰ in 2014 in Zhejiang Province. Mortality of children in rural regions decreased more remarkably than that in urban regions. *Chinese Statistic Year Book* revealed that in urban regions of China mortality of newborns, infants and children under five reduced from 7.5, 9.1 and 10.7 ‰, respectively in 2005 to 3.9, 5.2 and 5.9 ‰, respectively in 2012. Mortality rate in urban regions of China decreased more remarkably than that in rural regions; urban-rural inequity increased.

Comparing with data country-wide, Zhejiang province has achieved great effects in improving urban-rural equity of children’s survival. Urban-rural disparity maybe reduced due to the following policies: 1) Zhejiang Province was in the top-list to improve the balance between urban and rural development; 2) After new medical reforming implemented in Zhejiang province, the government increased financial investment for developing basic health care facilities and improved children’s health-care capacities in rural regions; 3) Improved medical care for children in rural regions improved health care access for children in rural regions.

### Still remarkable inequity of children’s survival in migrant population and children with native residency

China is now facing new challenges under rapid urbanization. The number of children from rural regions in urban areas increased under urbanization. As special population, migrant children in urban regions have more healthcare and survival problems. A report from Bangladesh revealed that mortality of migrant children under five in urban regions was 79% higher than that in overall urban children and 44% higher than that in rural regions [23]. Zhejiang province has a large population of migrant workers. In 2010, migrant children in Zhejiang Province reached 2 million, being in the top three of China. Mortality rate of migrant children was higher, which affects overall survival of children in urban regions.

Health inequity between children from migrant population and local registry has become a big challenge to public health in China. Our study revealed that mortality of migrant newborns reduced from 15.64 ‰ in 2005 to 7.96 ‰ in 2014. This study revealed that the problems of healthcare inequity remain in the following areas: 1) lack of surveillance in migrant population, children death due to accidental injuries was high; 2) access to healthcare in migrant population was limited due to low family income; and 3) Basic medical insurance in local areas was not applicable in hospitals in urban regions, and current household registration system in China had limited access to public health resources for migrant population in urban areas.

### Regional economical level and children’s mortality

The scatter diagram of this report revealed a negative relation between regional per capita GDP and mortality in children. After stratification on regions, mortality under five among the regions had significant difference, which was consistent with other report from China [24]. Population of economical deprivation should be a focused population requiring intervention to improve equity due to regional economical difference. Population of economical deprivation had low access to health care resources. Government should take measures to improve health care resources in economical deprivation population and regions.

Interestingly, our results also showed that regional economical level is not the sole factor influencing children’s mortality. Mortality in newborns and infants regions with middle economical level was higher than those in regions with low economical level. Therefore, regional economical development can improve children’s survival but not the only social determinant. In regions under economical deprivation, children’s mortality rate still can be reduced through improving education in mothers, taking optimal and economical interventional measures including maternal and children health care intervention, reasonable medical resources allocation and improving equity of health care, etc. Lass et al. revealed that community-based interventions integrating strategies such as home visiting and counseling can help to reduce fetal and neonatal mortality in low- and middle-income countries [25]. Therefore, formulating scientific health-care policy is the key factor for improving children’s survival.

The concentration curve of mortality rate of children under 5 years was near to equity curve. Children’s mortality rate is much higher in the regions with much lower GDP level. Li et al. used CI to compare children’s survival in different regions; they found that CI of children’s mortality was between −0.1 and −0.09, which suggests that children’s deaths focused in region with low per capita GDP [26]. Liang et al. reported CI of children’ mortality in Shandong Province in 2011 was −0.1782 [27]. The CI in Zhejiang Province was lower than that reported in other provinces in China, which suggests well equity in child survival.

## Conclusions

Our study has provided us several hints on policy alterations. Target policy should be formulated to improve survival level of migrant children and children of economical deprivation in China. One is registration reformation: we should eliminate the hierarchical differences in migrant population, and include migrant population into health care surveillance of local government; increase the equity access to health care resources for migrant population, educate mothers of the migrant population and provide more social supports for these families; and establish a supporting network to this particular population. Second is to focus on population of economical deprivation. Government should provide free maternal and children’s health care services for this population. Central government and local government should expand financial investment on public health resources, achieve regional health care sharing, expand the scope of the long-distance medical reimbursement, and reduce the economic burden of the migrant population and medical cost.

## Abbreviations

CC: Concentration curve
CI: Concentration index
GDP: Gross domestic product
